# The Effects of Winter Recreation on Alpine and Subalpine Fauna: A Systematic Review and Meta-Analysis

**DOI:** 10.1371/journal.pone.0064282

**Published:** 2013-05-15

**Authors:** Chloe F. Sato, Jeff T. Wood, David B. Lindenmayer

**Affiliations:** 1 Fenner School of Environment and Society, The Australian National University, Canberra, Australia; 2 ARC Centre of Excellence for Environmental Decisions, The Australian National University, Canberra, Australia; Stockholm University, Sweden

## Abstract

The ski industry is often perceived as having a negative impact on sensitive alpine and subalpine communities. However, empirical evidence of such impacts is lacking. We reviewed the available literature from the last 35 years to quantify the reported effects of winter recreation on faunal communities. Overall, using one-sample binomial tests (‘sign tests’) we found that the effects of all types of winter recreation-related disturbances (i.e. ski runs, resort infrastructure and winter tourism) were more likely to be negative or have no effect, than be positive for wildlife. More specifically, in Europe, where the majority of the available research was conducted, the impacts of winter recreation were most often negative for fauna. In terms of specific taxa, birds and to a lesser extent mammals and arthropods, responded negatively to disturbance. Results from our meta-analysis confirmed the results from our binomial tests. Richness, abundance and diversity of fauna were lower in areas affected by winter recreation when compared with undisturbed areas. For most regions and taxa, however, empirical evidence remains too limited to identify clear impacts of winter recreation. We therefore conclude that the majority of ski resorts are operating in the absence of knowledge needed to inform effective strategies for biodiversity conservation and ecologically-sound management. Thus, there is an urgent need for more empirical research to be conducted throughout this increasingly threatened ecological community, especially given the indication from the available literature that fauna often respond negatively to winter recreation.

## Introduction

Alpine ecosystems are significant for biodiversity [1], [2], [3] but only cover between 5.2–7.9% of the Earth’s terrestrial landmass (excluding the Antarctic landmass; [4], [5]). Many alpine and subalpine environments support highly endemic communities of taxa [6], [7] such as reptiles [8], birds [9], [10] and invertebrates [11], [12]. These ecosystems are also thought to be sensitive to human development [13], [14], [15], [16], domestic livestock grazing [17], invasive species [18] and intensive wildfires [19]. In addition, these environments are expected to be significantly affected by climate change [20], [21]. Under a conservative scenario of only 1°C global temperature increase, some authors predict a 150 m retraction of the snow line up the mountains [22], [23], [24]. Severe global warming scenarios (IPCC scenario A1B; [20]) predict that global temperatures may increase by 1.5–2.4°C by 2050 [20], [24]. This could see snow lines retract by as much as 220–360 m upslope (based on figures provided by [20], [22], [23], [24]). Indeed, snow line retractions may be even more severe than predicted when other factors such as snow-pack variability, North Atlantic Oscillations and El Niño/La Niña are considered [25], [26].

The ski industry is already implementing climate adaptation strategies (e.g. artificial snow making and the introduction of high capacity ski lifts that can access higher elevation ski runs) to increase longevity of winter tourism activities [20], [22], [27]. These adaptation strategies may adversely affect fauna [20] by limiting the area of undisturbed habitat available for endemic species, especially those sensitive to human disturbances (e.g. Mountain Pygmy Possum, *Burramys parvus*, in south-eastern Australia [28], [29]).

Effective management and conservation of alpine and subalpine systems requires high quality empirical information to guide policy and on-the-ground management interventions. Yet, major knowledge gaps remain in many areas ranging from the biology of alpine organisms [30], [31] to the effects of stressors, such as human-induced environmental change, on populations and communities [11], [32], [33], [34].

To date, there has been no systematic review of key threatening processes influencing biodiversity in alpine-subalpine systems, particularly in regards to immediate and direct human-driven impacts potentially arising from tourist resort development and extension. Yet, this information is critical to guiding effective management and evidence-based policies. To address this major knowledge gap, we have conducted a targeted and detailed systematic review and meta-analysis of the global literature to quantify the impacts of ski tourism and ski resort infrastructure on alpine-subalpine wildlife. To this end, we asked the following key questions:

1. Which taxa appear to be most heavily affected by alpine and/or subalpine development?
2. Are there regional patterns in biotic response with some areas experiencing greater impact than others?
3. Is the nature of the impact consistent across different winter recreation-related disturbances?

## Methods

For the purposes of this review, we classify alpine and subalpine areas using the globally applicable definitions provided by Löve (1970; [5]). She defines an alpine area as the zone above the limit of physiological tree growth (the treeline) and a subalpine area as the natural belt that lies between the treeline (at its upper limit) and the closed montane forest (at its lower limit).

We focused this review on studies that generated empirical data on the impacts of human disturbances on terrestrial vertebrate and invertebrate taxa of alpine and subalpine areas around the world. Human disturbances included ski-related developments (such as roads, buildings and ski-lifts; henceforth termed ‘Resort Infrastructure’), direct mountain management and modification (such as slope and snow grooming; henceforth termed ‘Ski Runs’), and direct human impact from winter recreational sports (including snow-shoeing, skiing, snowboarding and over-snow vehicle use; henceforth termed ‘Winter Tourism’).

### Literature Search

**Figure 1:**
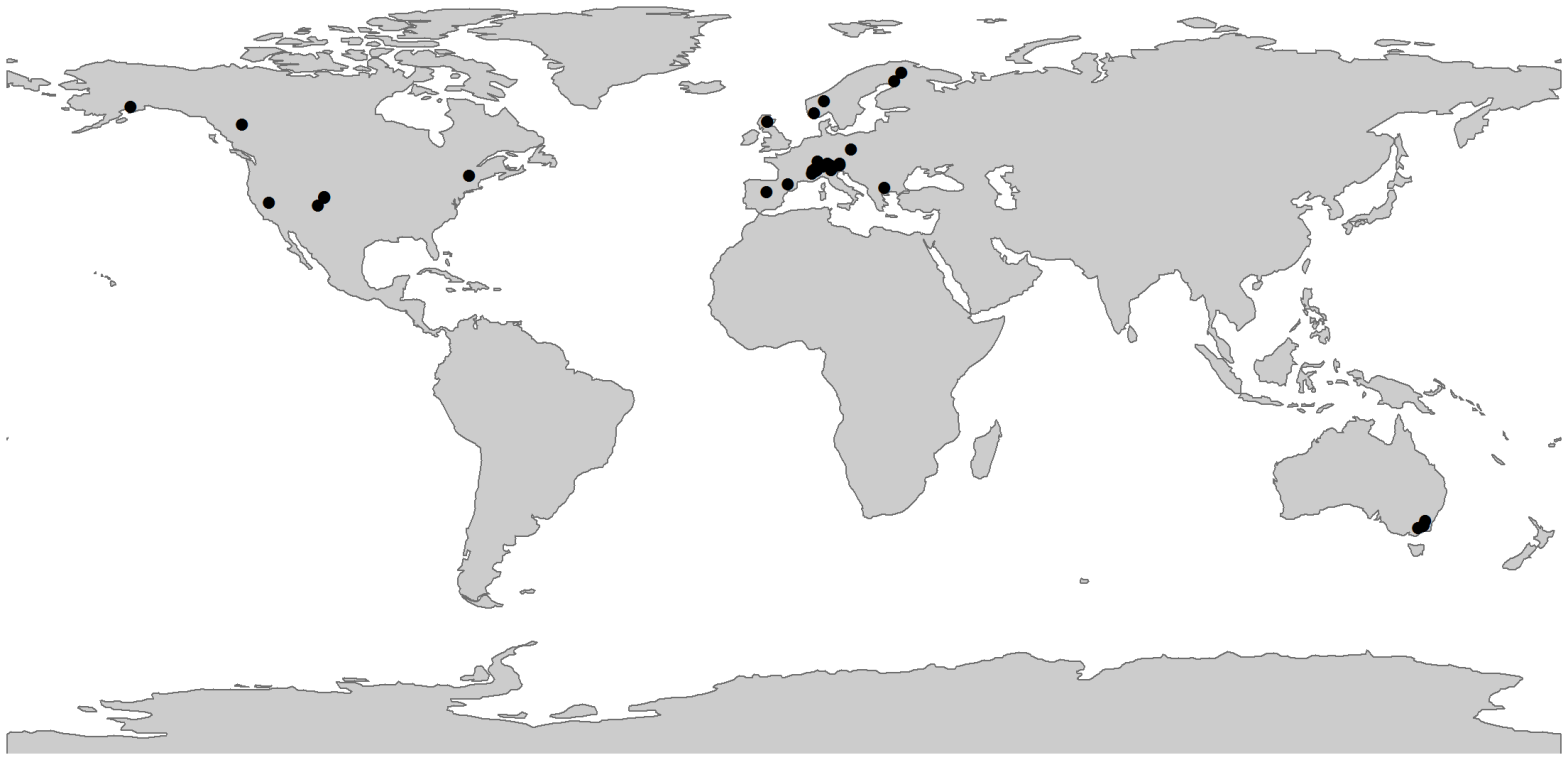
Global distribution of studies (n = 41) that investigated the effects of winter recreation on wildlife.

Due to the broad, geographical nature of our review (see Figure 1), obtaining all the “grey” literature on the impacts of ski resorts on alpine and subalpine fauna was not feasible. As such, we only used the peer-reviewed primary literature as identified via electronic databases for our analyses.

We searched four major electronic databases - Web of Science (1945-present), Zoological Record Plus (1978-present), ProQuest: Science and Technology Databases (1967-present) and CAB Abstracts (1973-present) - on the 16^th^ April 2012 using the following search string: ((ski slope* OR ski lift* OR ski run* OR ski piste* OR piste* OR ski resort* OR “ski develop*”) OR (“snow* sport*” OR ski sport* OR winter sport* OR “winter recreation”) OR (“winter tourism” OR “ski tourism” OR “nature tourism” OR snow tourism) OR (“alpine habitat fragment*” OR “alpine modification” OR devegetated matrix) OR (“subalpine habitat fragment*” OR “subalpine modification” OR devegetated matrix) OR (snow AND alps)) AND (fauna* OR (bird* OR avifauna*) OR mammal* OR (reptil* OR lizard*) OR (frog* OR amphib*) OR (invertebrat* OR insect*)).

We used different combinations of search terms based on the requirements or limitations of each database. No constraints on year of publication or language of publication were imposed on the database searches. We also examined additional, relevant articles collected opportunistically over the previous year.

### Extraction of Data for Analysis

**Figure 2:**
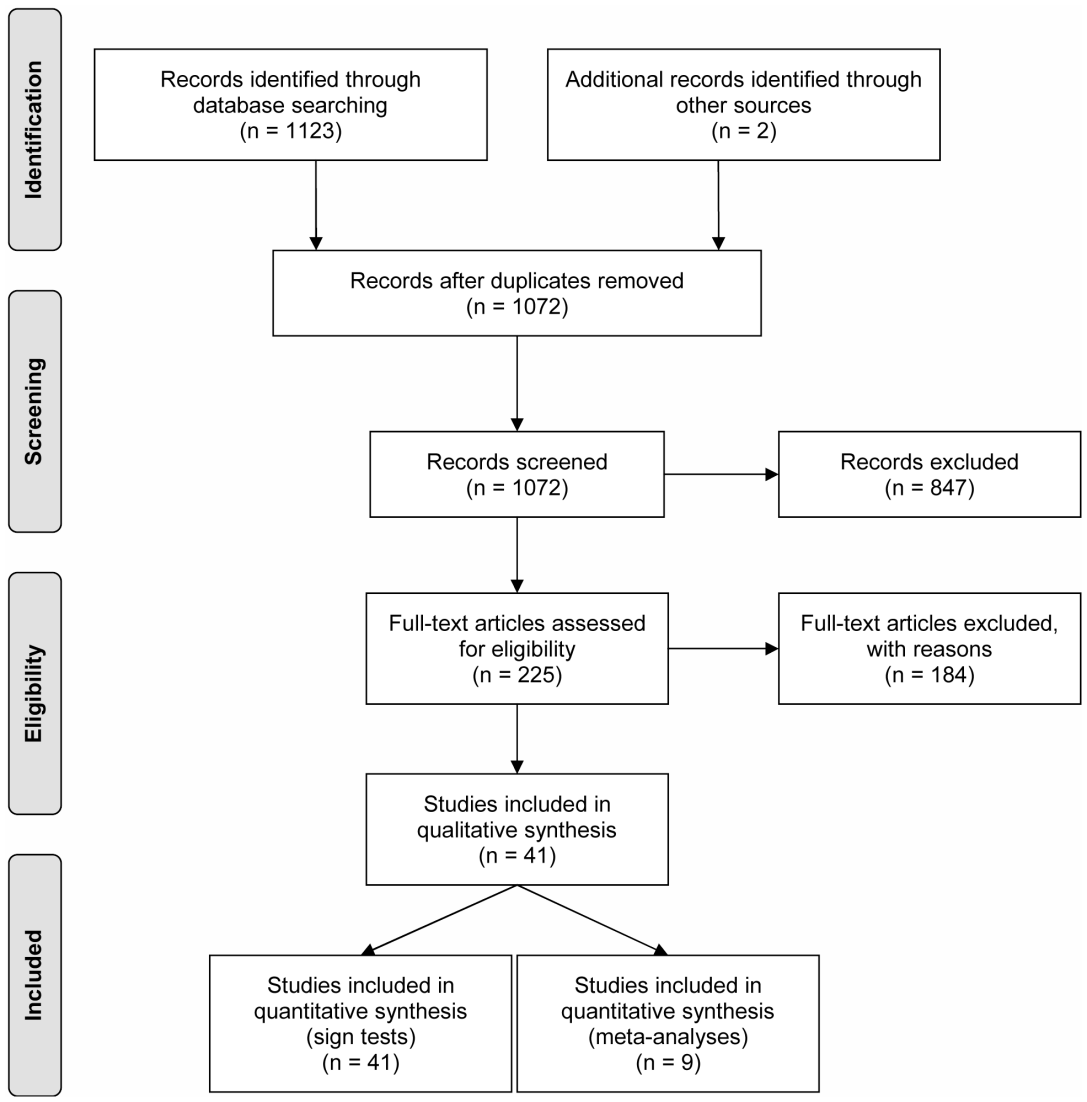
PRISMA Literature Search Flow Diagram.

Our systematic literature search was designed to find studies related to the effects of ski resort infrastructure and winter recreation on wildlife. To ensure that we only included articles in the review specifically related to this topic, we used two levels of screening. At the first level of screening, we read titles and abstracts, excluding articles that did not satisfy at least two of the following criteria: 1) Focus on fauna, 2) Mention ski resorts, ski infrastructure or winter sports, 3) Concentrate on alpine or subalpine environments. Full text articles were obtained for all the articles that passed the first level of screening. At the second level of screening we read entire articles, excluding those that did not: 1) include original research focussing on effects of winter recreation-related disturbances on fauna, 2) contain usable, empirical data, or 3) provide statistical analysis of data. At each level of screening, we recorded the number of articles identified and the number of studies included and excluded according to the Preferred Reporting Items for Systematic Reviews and Meta-Analyses (PRISMA) Statement (see Figure 2).

For each of the 41 papers included in the final analysis (see Table 1, Reference List S1), we posed the following questions. (1) In which country was the study conducted? (2) In what year(s) was the study conducted? (3) What taxonomic group was examined? (4) What type(s) of winter recreation-related disturbance was examined? (5) What measurements were taken to determine the effect of winter recreation? (6) What overall effect on the fauna was observed (as concluded by the author)? (7) What specific effect on fauna was observed for each measure recorded in the study? (8) Were any management recommendations included? We also assigned each paper an experimental design/data quality category (I–IV), as outlined in Table S1. We show the proforma used to extract information from retained articles in Table S1 and PRISMA Checklist in Checklist S1.

**Table 1:** Details of the 41 studies* investigating the impacts of winter recreation on alpine and subalpine fauna.

Author	Country	Taxa
Amo et al. (2007)	Spain	Reptile
Arlettaz et al. (2007)	Switzerland	Bird
**Ballenger & Ortega (2001)	USA	Bird
Baratti et al. (2000)	Italy	Arthropod
Braunisch et al. (2011)	Switzerland	Bird
Broome (2001)	Australia	Mammal
**Caprio et al. (2011)	Italy	Bird
Caravello et al. (2006)	Italy	Annelid
Foissner et al. (1982)	Austria	Protozoan
Goldstein et al. (2010)	USA	Mammal
Goodrich & Berger (1994)	USA	Mammal
Green (2000)	Australia	Mammal
Hadley & Wilson (2004a)	USA	Mammal
Hadley & Wilson (2004b)	USA	Mammal
Haslett (1991)	Austria	Arthropod
Haslett (1997)	Germany	Arthropod
Jokimaki et al. (2007)	Finland	Bird
**Keßler et al. (2012)	Austria & Germany	Arthropod
Krebs et al. (2007)	Canada	Mammal
Kübelböck & Meyer (1981)	Austria	Annelid
**Laiolo & Rolando (2005)	Italy	Bird
Lüftenegger et al. (1986)	Austria	Protozoan+Nematode
Mansergh & Scotts (1989)	Australia	Mammal
**Mincheva et al. (2009)	Bulgaria	Nematode
Morrison et al. (1995)	USA	Mammal
**Negro et al. (2009)	Italy	Arthropod
**Negro et al. (2010)	Italy	Arthropod
Nellemann et al. (2000)	Norway	Mammal
Patthey et al. (2008)	Switzerland	Bird
Reimers et al. (2003)	Norway	Mammal
**Rolando et al. (2007)	Italy	Bird
Sanecki et al. (2006)	Australia	Mammal
Shine et al. (2002)	Australia	Reptile
**Strong et al. (2002)	USA	Arthropod
Szymkowiak & Gorski (2004)	Poland	Arthropod
Thiel et al. (2007)	Germany+France	Bird
Thiel et al. (2008)	Germany	Bird
Thiel et al. (2011)	Germany+Switzerland	Bird
Ukkola et al. (2007)	Finland	Mammal+Bird
Watson (1979)	Scotland	Mammal+Bird
Watson & Moss (2004)	Scotland	Bird

* Complete reference details provided in Reference List S1;

** indicates studies included in the meta-analyses.

### Reporting of Results

We collated the information extracted from each paper in qualitative tables and presented these results using simple tables and bar charts. As 20 different measures of biotic response were reported across the included studies, we recorded the overall effect identified by each individual paper, but also pooled logical subsets of biotic response measures to create four composite categories. In doing so, we could analyse the effects of winter recreation-related disturbances on specific biotic responses of fauna. The four composite categories analysed were:

1. *population and community descriptors*: measures used to describe a population or community of animals (e.g. abundance, diversity, richness and community composition);
2. *population viability measures*: measures that may contribute to the persistence and viability of populations (e.g. breeding success, recruitment and survival);
3. *fitness measures*: measures that may contribute to the survival of an individual (e.g. body condition, parasite load and sprint speed); and
4. *‘other’ measures*: measures that did not fall under one of the above categories (e.g. habitat use, frequency of occurrence and predicted presence).

Examples of the types of results we recorded as ‘positive effect’, ‘negative effect’ and ‘no effect’ for each of the composite categories are provided in Table S2.

To test the hypothesis that human-generated disturbances were equally likely to have a positive or negative effect on fauna, we used data from studies that demonstrated clear positive or negative effects on fauna and omitted from analysis ambiguous results (i.e. ‘no effect’ or variable results). We calculated the proportion of studies that had a positive effect on the focal species or focal community and used one-sample binomial tests (‘sign’ tests; [35]) to assess whether this proportion was significantly different from 0.50. We performed separate one-sample binomial tests for each of the data sets (continents, taxa, and specific types of winter recreation-related impacts). Data sets with sample sizes of less than six were omitted from our analysis as the statistical power to detect significant differences is likely to be inadequate below this level [36].

The use of one-sample binomial tests allowed us to statistically test broad hypotheses about the effects of winter recreation on fauna despite the variability in the measures reported among included articles [35]. We also conducted a meta-analysis to determine the effect of winter recreation on fauna. We found that only three measures – richness (n = 8), diversity (n = 6) and abundance (n = 7) - had a sufficient number of studies (five or more) that provided the required information (means, standard deviations and sample sizes) to calculate an overall effect size using weighted Hedges’ *d* [37]. To make these effect sizes ecologically meaningful, we first standardised the reported data so that each study only yielded means and standard deviations for specific taxonomic assemblages (e.g. birds, mammals, arthropods etc.) Thus, for those studies where information was provided for individual species or individual study areas, we combined site-level or species-level means and standard deviations for each measure according to formulae provided by Borenstein et al. [35,p.222]. We then calculated the effect sizes for each study using weighted Hedges’ *d*, as well as an overall effect size of winter recreation for each measure by combining the effect sizes across studies (see [37,p.16]). We assessed winter recreation as having a statistically significant effect on a given measure if the 95% confidence interval (CI) did not overlap zero. We conducted all meta-analyses using Genstat 15 (VSN International Ltd).

## Results

Our systematic literature search retrieved 1072 articles that were potentially relevant to the review. Of these, we found 847 abstracts to be unrelated to the impacts of winter recreation on wildlife and so we rejected them. We read the remaining 225 articles in full and found a further 184 did not meet our inclusion criteria. Thus, we retained 41 articles for our final analysis (Figure 2).

**Figure 3:**
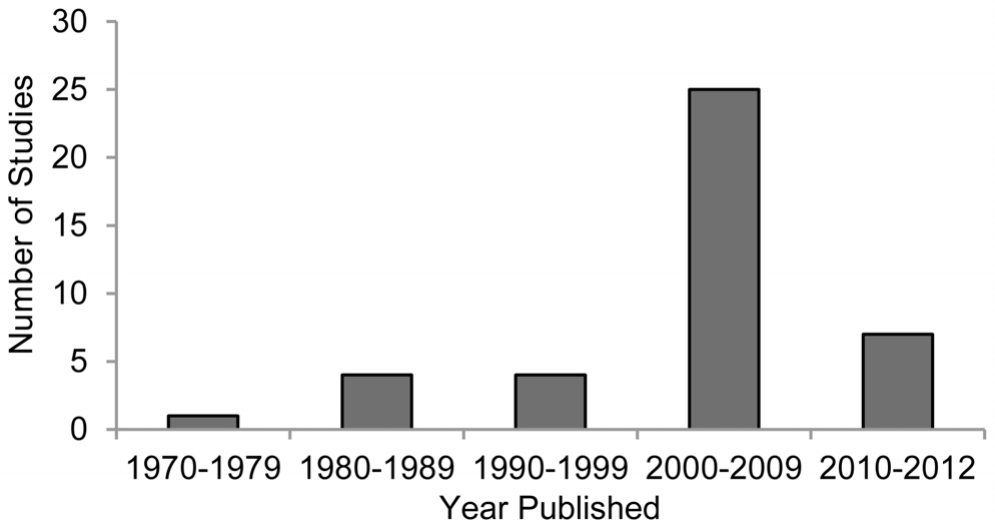
Publications by decade. The number of studies (n = 41) published by decade, investigating the impacts of winter recreation on fauna from 1970 to mid-2012.

The 41 articles included in our analysis were sourced from 25 different journals. The majority were published in *Biological Conservation* (14.6%), *Journal of Wildlife Management* (14.6%), *Journal of Applied Ecology* (9.6%) and *Biodiversity and Conservation* (7.3%). All other journals published two or fewer articles related to the impacts of winter recreation on fauna. In addition, the number of publications increased over time from just one during the 1970s to 25 during the period 2000–2009 (Figure 3). Seven studies have been published since 2010 (Figure 3).

Generally, the experimental design and data quality of papers was high according to the criteria in Table S1. Most studies (34/41) were assigned to category one or two (i.e. controlled studies with adequate replication). The remainder fell into category three (7/41; replication was not adequate). No study fell into category four (i.e. control was absent).

### Geographical Area

Most studies were conducted in Europe, predominantly in Italy, Austria and Germany (Table 2). The remainder were conducted in North America (primarily in the USA) and Australia (Table 2). No studies were completed in South America, Asia or Africa. The proportion of available studies in the peer-reviewed literature conducted in Europe and North America approximates the global proportion of ski resorts in these areas i.e. European studies make up 68% of included articles and European ski areas represent approximately 62% of all ski areas [38]. In comparison, Asia and South America were clearly under-represented in the peer-reviewed literature considering the proportion of ski areas located in these regions (Table S3). On the other hand, Australia was over-represented, contributing 12.2% of peer-reviewed studies but representing only 0.2% of all ski areas (Table S3).

**Table 2:** Numbers of published studies that investigated the impacts of winter recreation on wildlife.

Category			Total	Negative	No Effect	Positive
**Continent**						
	**Europe**		28			
		Austria*	5	8	6	2
		Italy*	7	11	8	3
		Switzerland*	4	4	–	–
		Bulgaria	1	–	1	–
		Spain	1	1	1	–
		Germany*	5	5	1	1
		Finland	2	1	2	2
		France*	1	1	–	–
		Norway	2	1	1	–
		Poland	1	–	1	–
		Scotland	2	2	2	1
	**North America**		8			
		USA*	7	6	4	4
		Canada*	1	1	1	–
	**Australia**		5	4	1	2
**Taxon**						
		Bird	14	13	7	2
		Mammal	15	11	8	5
		Reptile	2	1	1	1
		Arthropod	9	12	8	7
		Annelid	2	1	1	–
		Nematode	3	2	1	–
		Protozoan	2	4	4	1
**Biotic Measure**						
	**Population & Community** **Descriptor**					
		Abundance	11	13	5	2
		Biomass	2	1	1	–
		Density	9	7	7	3
		Diversity	4	3	3	1
		Richness	11	7	6	1
		Dominant Species	3	4	5	4
	**Population Viability Measure**					
		Breeding Success	1	1	1	–
		Number of Nests	1	–	–	1
		Distance Travelled	5	3	2	–
		Recruitment	1	1	–	–
		Survival	4	1	3	2
		Frequency of Abandonment	1	1	–	–
	**Fitness Measure**					
		Body Condition	1	1	–	–
		Parasite Load	1	–	1	–
		Sprint Speed	1	1	–	–
		Stress Hormone	3	3	–	–
	**Other Measure**					
		Habitat Use	3	3	2	1
		Frequency of Occurrence	1	1	–	1
		Predicted Presence	1	1	–	–
**Impact Type**						
		Ski Run	22	26	21	11
		Resort Infrastructure	10	8	6	4
		Winter Tourism	9	8	2	0

* indicates a country with a major ski market [38]. ‘Total’ refers to the total number of published studies. ‘Positive’, ‘No Effect’ and ‘Negative’ refer to the specific effect(s) reported in each study. Note that the total number of specific effects may not equal the total number of studies, as individual articles may have reported more than one effect (e.g. an effect for each individual species, functional group, biotic measure etc. studied).

In terms of management, Australian and North American studies provided recommendations in the majority of their publications (4/5 and 6/8 respectively), whereas European studies did so in only 60.7% of publications (17/28). No temporal bias was apparent in the provision of recommendations for each region (Table S4).

### Taxonomic Groups

**Figure 4:**
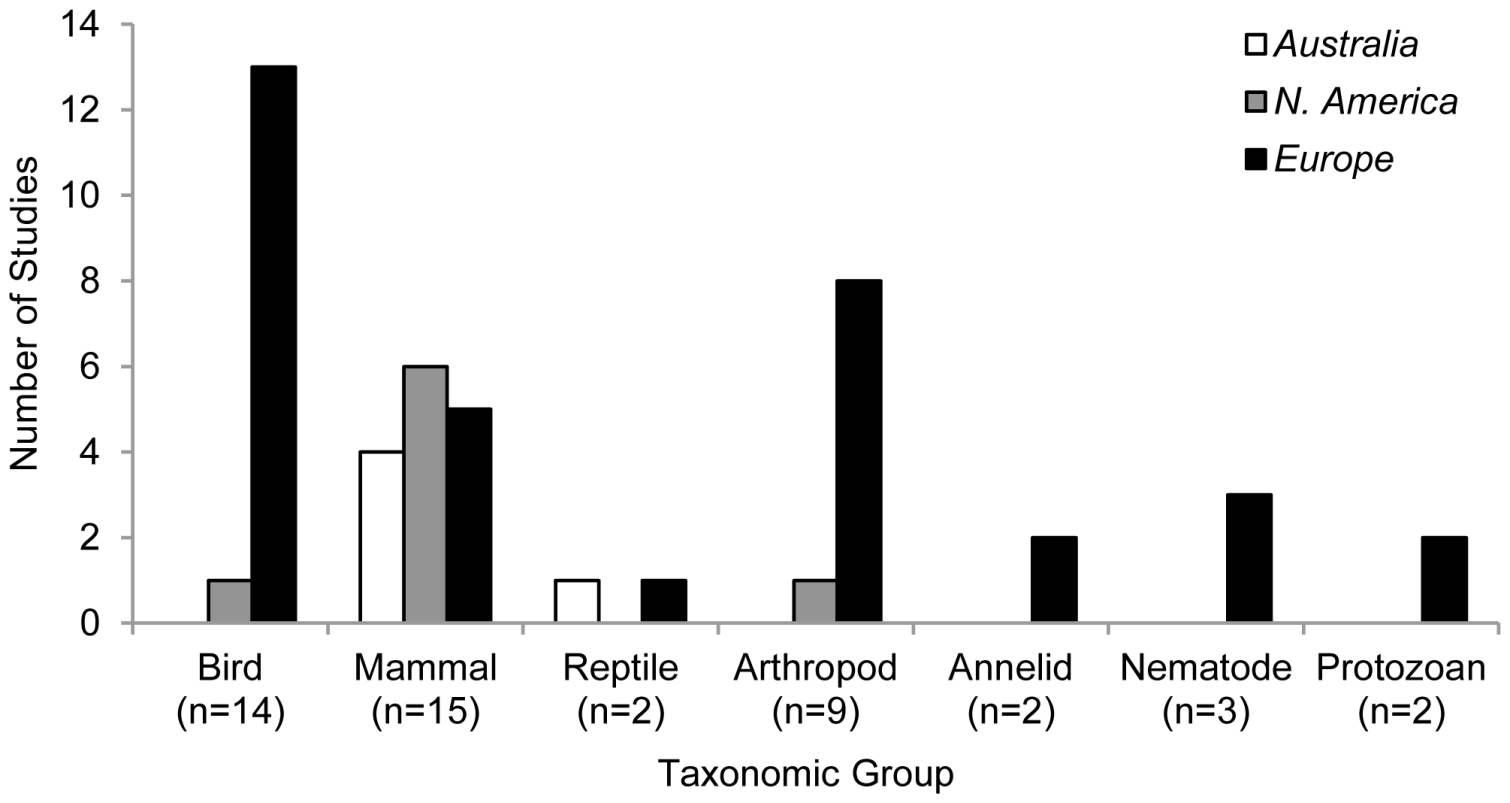
Number of studies (n = 41) investigating the impacts of winter recreation on different taxa across three continents. Four studies investigated multiple taxa, hence totals for taxonomic groups do not sum to the total number of studies analysed.

Just over one third of all studies investigated the impacts of ski infrastructure on mammals, with birds and arthropods also well represented in the literature. A smaller number of studies investigated other taxa. No studies focused on amphibians (Figure 4).

Regionally, there was variation in the taxonomic focus. Studies conducted in Europe investigated a diversity of taxonomic groups but the majority concentrated on birds and arthropods. In contrast, North American and Australian studies predominantly investigated mammals. Birds, reptiles and invertebrates were infrequently (or not) studied in these two regions (Figure 4).

### Effects of Winter Recreation

**Figure 5:**
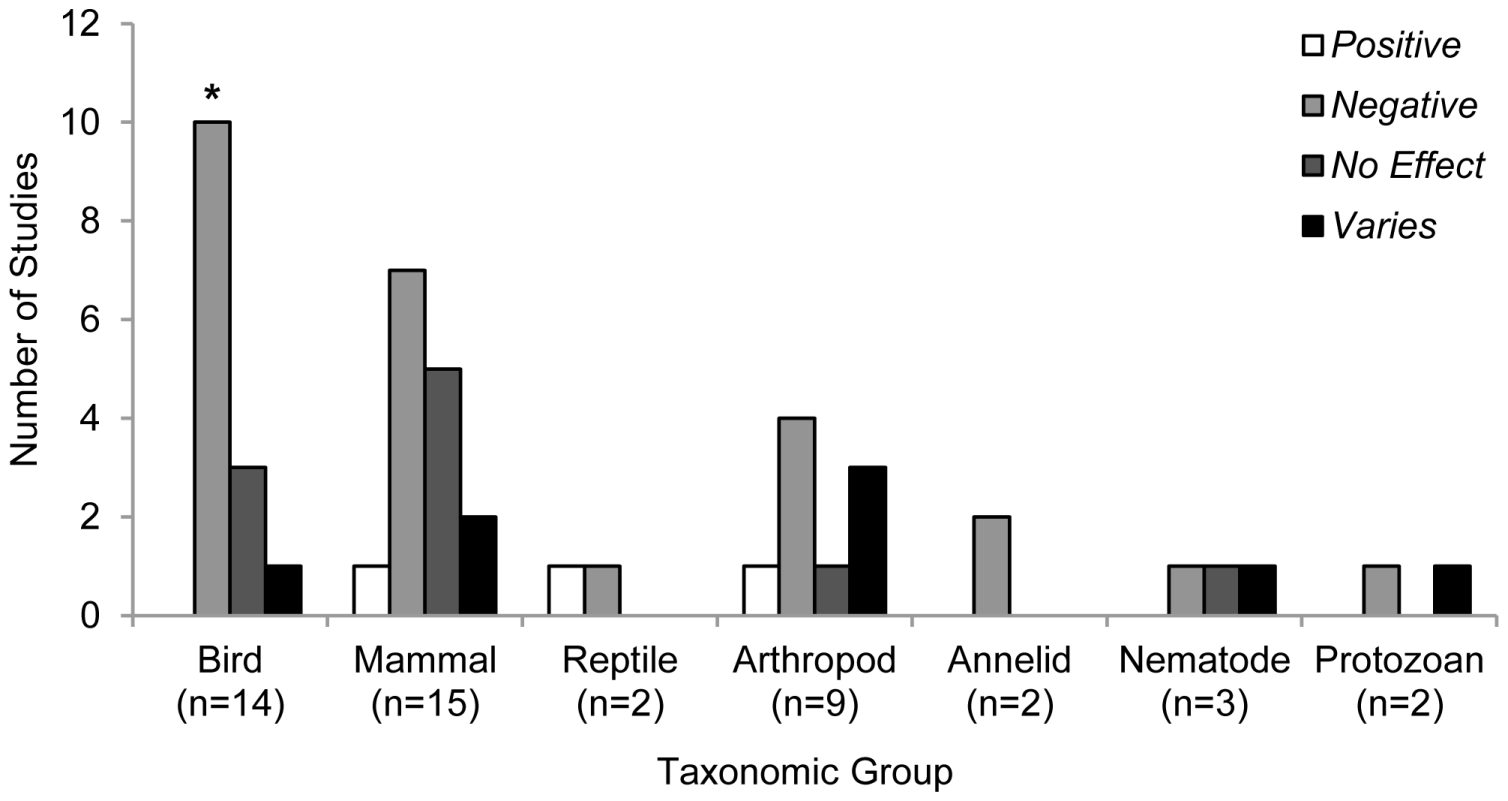
Number of studies (n = 41) investigating the effects of winter recreation on seven different taxonomic groups. **P<0.01 indicates a significant difference between the number of positive and negative effects reported.

**Figure 6:**
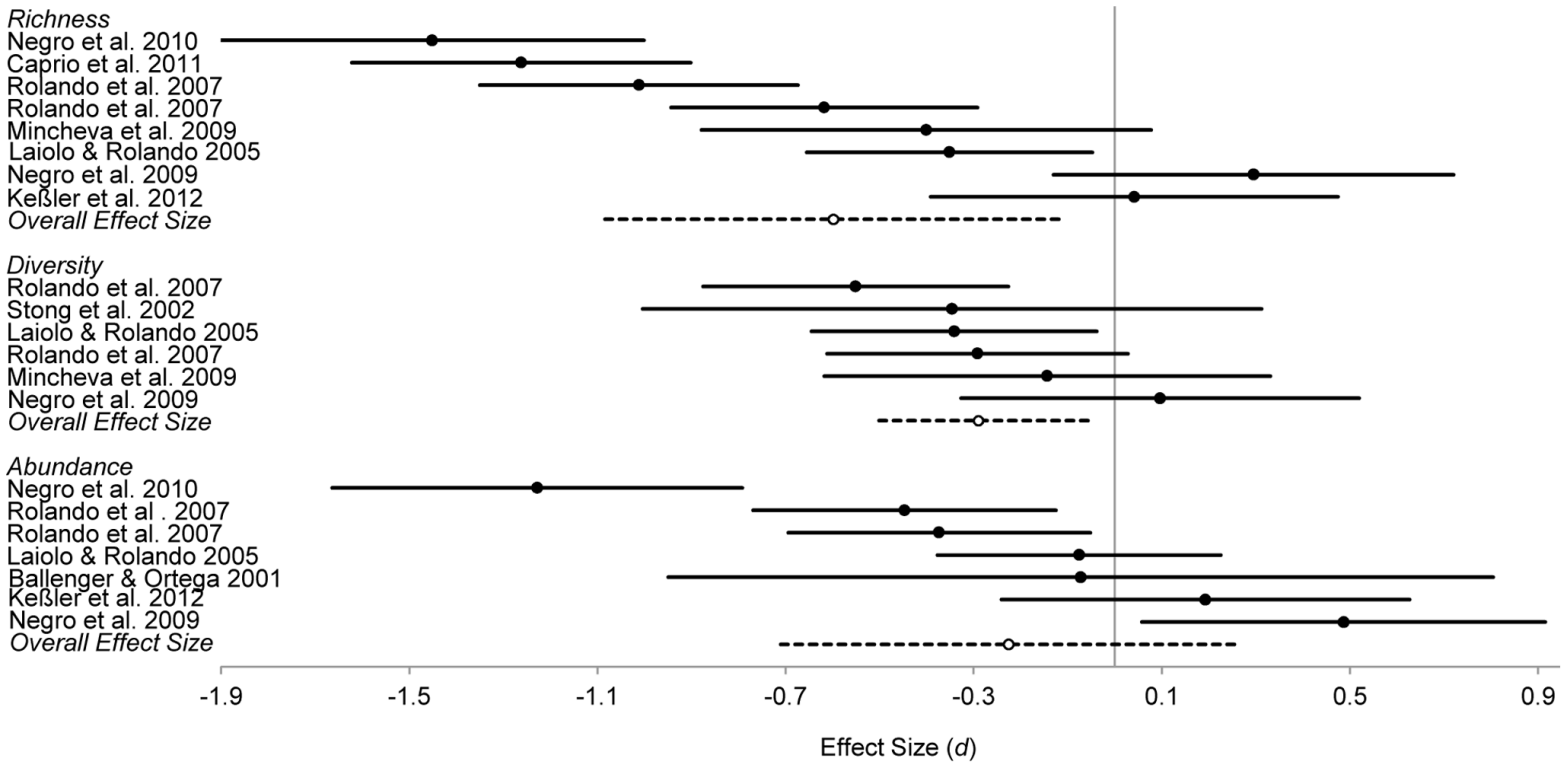
Overall and individual study effect sizes (±95% C.I.) of winter recreation on fauna (*d*). Weighted effect sizes were calculated for meta-analyses on measures of richness, diversity and abundance.

More than half the studies reported overall negative effects of human-generated disturbance on fauna in alpine and subalpine areas (P<0.001). Few studies reported overall positive effects (Figure 5). The remainder reported overall non-significant (‘no effect’) or variable effects (i.e. a combination of positive, negative and non-significant effects dependent upon species or measure taken). For three reported measures (richness, diversity and abundance), sufficient information was provided to conduct a meta-analysis. This analysis confirmed the significant negative impact that winter recreation has on the richness (*d* = −0.60, df = 7, 95% C.I.: −1.08 to −1.02; Figure 6) and diversity (*d* = −0.29, df = 5, 95% C.I.: −0.52 to −0.07; Figure 6) of alpine-subalpine fauna. Winter recreation also has a negative effect on the abundance (*d* = −0.23, df = 6, 95% C.I.: −0.70 to 0.25; Figure 6) of fauna, however this result was not significant.

When we analysed the effects of winter recreation by taxonomic group, we found that studies generally reported negative effects for winter recreation-related disturbances on birds (P = 0.002) and annelids; negative or variable effects on arthropods; and negative (P = 0.07) or non-significant effects on mammals. The results reported for reptiles, nematodes and protozoans were variable and did not show consistent trend patterns (Figure 5).

We also quantified how winter recreation affected biotic responses of different taxonomic groups. From 20 measures of biotic response reported across 41 studies, we pooled similar measures to collapse our data into four composite categories. These categories were: 1) population and community descriptors; 2) population viability measures; 3) fitness measures; and 4) other measures.

When composite categories were considered for each taxonomic group, protozoan (n = 5), nematode (n = 2), reptile (n = 2) and annelid (n = 1) sample sizes were too small for meaningful analysis. For all other taxonomic groups, population and community descriptors were the most commonly reported measures (Table 2). Winter recreation was often reported to have a negative effect on population and community measures for birds (P = 0.18) and arthropods (P = 0.30), but these were not significant results. For mammals, the effects were almost equally likely to be positive or negative (P = 0.73). However, when composite categories were pooled, winter recreation was significantly more likely to have a negative impact on birds (P = 0.004). Negative impacts were also common for mammals and arthropods but these results were not significant (P = 0.29 and P = 0.21 respectively; Table 2).

**Figure 7:**
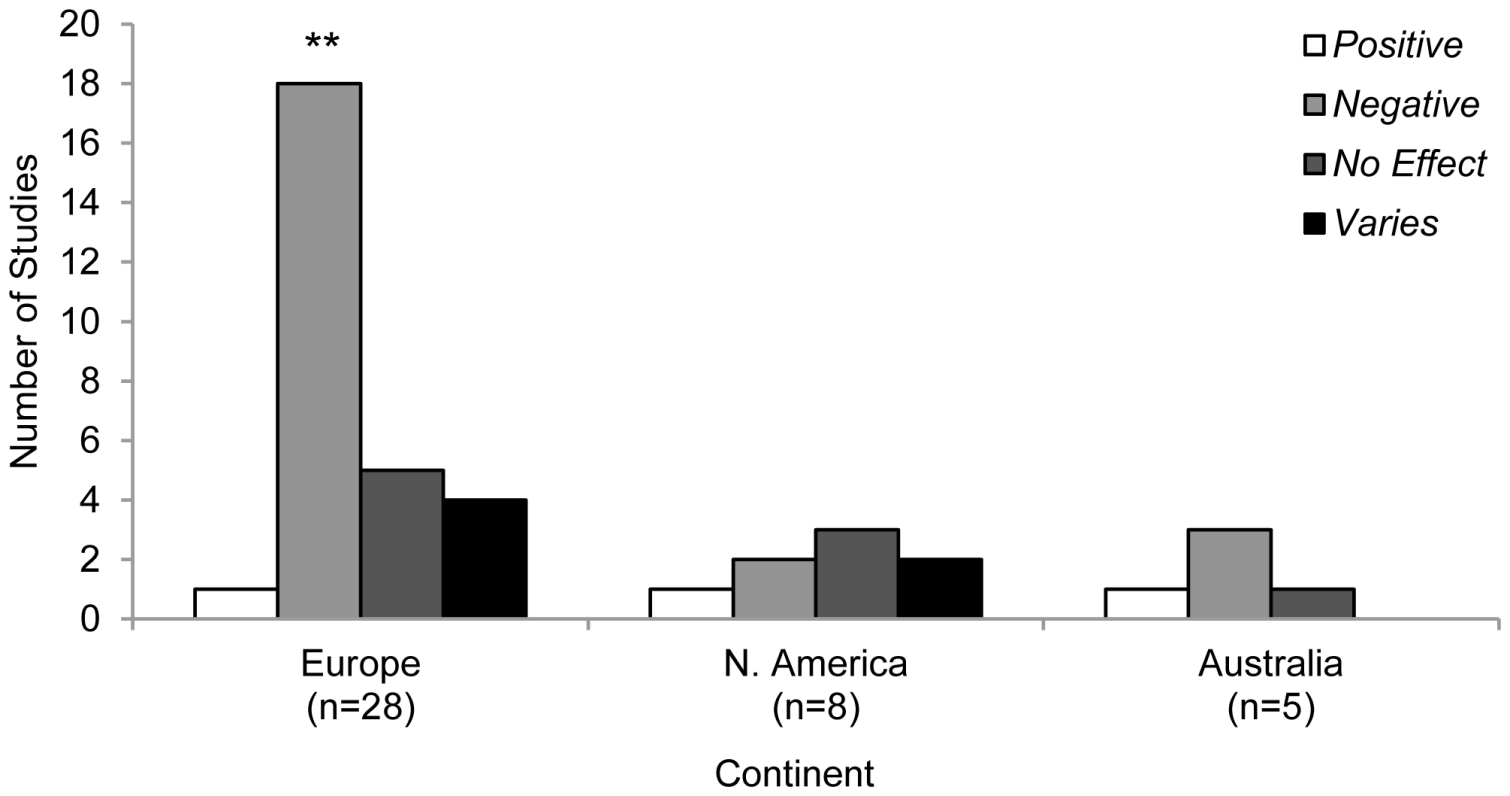
Number of studies (n = 41) investigating the effects of winter recreation on fauna across three continents. **P<0.01 indicates a significant difference between the number of positive and negative effects reported.

Regionally we found that, of the continents where the effects of skiing and resort infrastructure were investigated, European studies were more likely to report negative impacts of human-generated disturbance on fauna (P<0.001), as were Australian studies (Figure 7). However, data from Australian studies were too sparse for statistical analysis. North American studies reported a range of effects but, again, data was too limited for statistical analysis.

We also considered how biotic responses to winter recreation varied by region. European studies primarily reported population and community measures, while Australian studies predominantly reported population viability measures. North American studies reported these measures evenly (Table 2). When composite categories were pooled for each region, disturbances were significantly more likely to be negative in Europe (P<0.001). The effects of disturbance were also often reported to be negative in North America, but this trend was not significant (P = 0.344). In contrast, Australian studies reported positive effects almost as often as negative effects (P = 0.687; Table 2).

**Figure 8:**
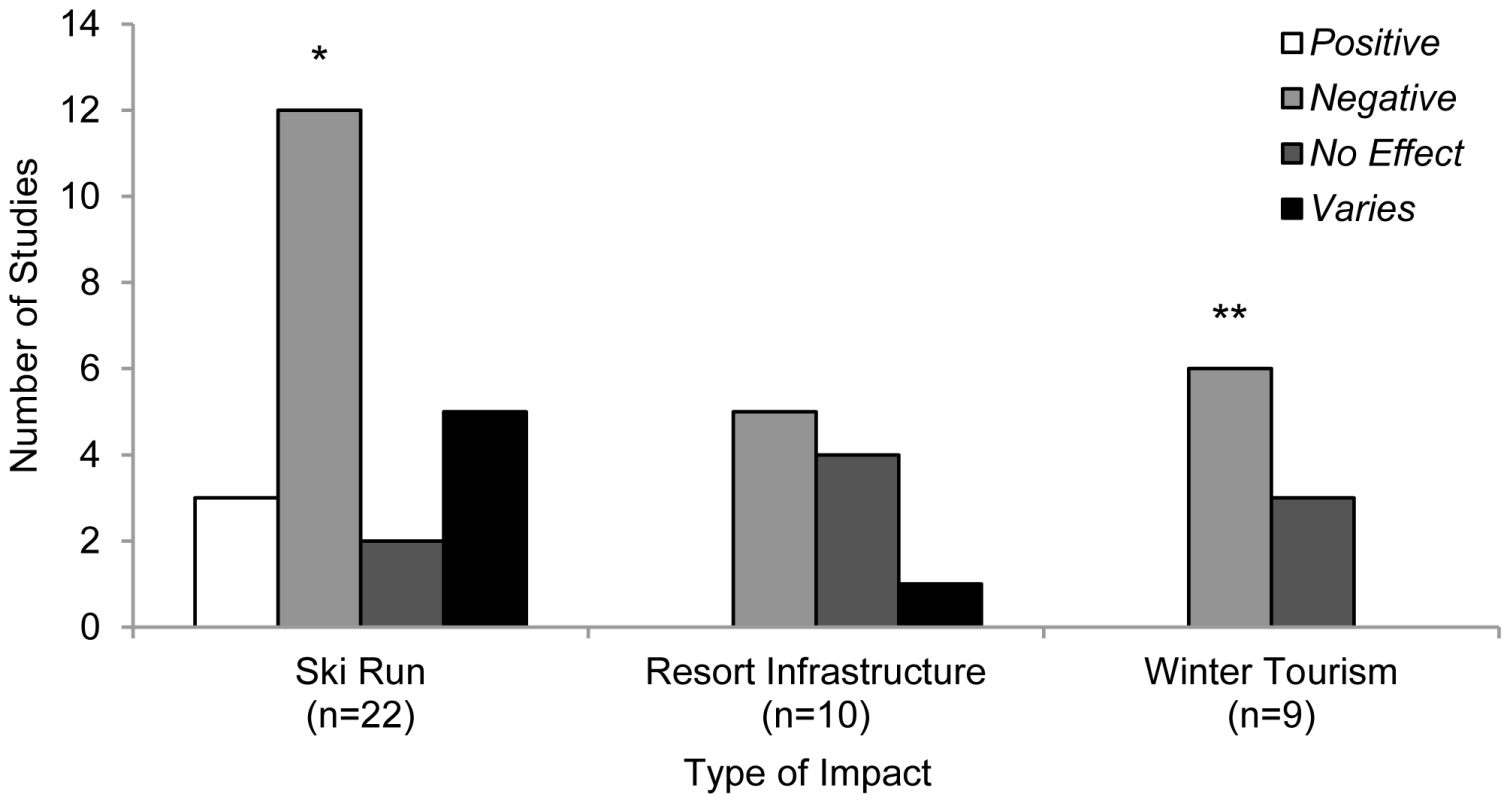
Number of studies (n = 41) investigating the effect of three different types of winter recreation-related disturbance on fauna. *P<0.05, **P<0.01 indicate a significant difference between the number of positive and negative effects reported.

Finally, we analysed the effects of specific winter recreation-related disturbances on wildlife. We found that the effects of ski runs on fauna were well represented in the literature, while the effects of resort infrastructure and winter tourism were less frequently studied (Figure 8). Both ski runs and winter tourism were significantly more likely to have a negative impact on fauna (P = 0.020 and P = 0.004 respectively; Table 2). Resort infrastructure also had a predominantly negative effect, but this was not significant (P = 0.388; Table 2).

## Discussion

The negative impacts of winter recreation on fauna have often been highlighted in the alpine-subalpine literature [39], [40], [41], [42]. However this article is the first systematic study of that literature. We addressed three key questions with respect to ski developments and impacts, and from these questions found that disturbances arising from winter recreation are more likely to have negative or non-significant impacts than have positive impacts on wildlife, regardless of taxonomic group, geographical region or specific type of ski modification. But more empirical studies are urgently required as many countries and taxa that are impacted by ski resorts are inadequately represented in the peer-reviewed literature.

The notion that ski resorts and their associated disturbances are likely to have negative impacts on biodiversity in alpine and subalpine areas is not surprising, as the construction of resort infrastructure requires the removal and modification of vegetation, as well as significant fragmentation of habitat over a small area [6]. While habitat fragmentation can have a variety of impacts on fauna [43], it is likely that habitat removal has the greatest effect on species with specialised habitat requirements [6]. This is particularly relevant to alpine-subalpine endemics of conservation concern such as the Mountain Pygmy Possum, *Burramys parvus*, in Australia and Raetzer’s Ringlet, *Erebia christi*, in Europe [29].

However, for many species and taxa there is little or no information available in regards to the impacts of ski resorts and winter tourism. The negative effects of disturbances arising from winter recreation (such as decreased species richness or increased levels of stress hormones) were most frequently reported for mammals, birds and arthropods. These taxonomic groups are relatively well studied in the available literature and some species (and groups) have even been suggested as suitable bioindicators for alpine and subalpine ecosystems, such as the Black Grouse (*Tetrao tetrix*; [44], [45]), syrphid flies [14], [46] and grasshoppers [33], [47]. As the impact of skiing is more likely to be negative on these prospective bioindicators, there is the potential that the effects of the ski industry are more extensive in alpine and subalpine environments than has been documented to date. This is because bioindicators tend to possess a moderate tolerance to disturbance, so it is likely that rare or sensitive species will already have been negatively affected by human-generated habitat modifications before the bioindicators show a response [48]. In addition, as many taxa remain poorly studied in areas affected by ski resorts, the suitability of bioindicators in representing general responses of fauna to disturbance in alpine-subalpine ecosystems cannot be adequately tested, nor can the lack of a response by a bioindicator be taken to mean that the community is not being adversely affected. Thus, more research is needed to improve our understanding of community interactions and species-level responses to disturbances in these environments.

We also need to improve our regional understanding of the impacts of winter recreation on wildlife. Most studies about the impacts of ski resorts on fauna come from Europe, with a smaller number of contributions from North America and Australia. Given that there are around 6000 ski areas located in more than 70 countries across the globe [38], many alpine and subalpine faunal communities around the world have not been studied. Thus, there is no regionally-relevant information about the effects of winter recreation on wildlife in many parts of the world. This is a concern as some countries with medium-sized developments (i.e. ski areas with 10 or more ski lifts) or large ski developments (i.e. ski areas with one or more major resorts) such as Andorra, New Zealand, Japan, China and Argentina [38] have not been the focus of any empirical research published in the peer-reviewed literature. Yet, three of these countries have been listed as global biodiversity hotspots (New Zealand, Japan and China; [49]). In addition, according to the IUCN red list, many of these nations contain species of conservation concern in ski resort areas [29].

Finally, we must recognise that all types of winter recreation-related disturbance are more likely to have negative than positive impacts on fauna (particularly ski runs and winter tourism). However, investigations into the effects of resort infrastructure and winter tourism still need more attention, as published studies are uncommon. Identifying the effects of specific winter recreation-related disturbances is important as it will help to target management strategies for ski resorts. For example, if direct human provocation of fauna during winter has a negative effect on the fitness of individuals or populations, then an effective management strategy would involve the reduced access of tourists to areas where nests, dens or hibernacula are located. Other strategies such as modifying grooming techniques or reducing the extent of artificial snowing may not significantly improve the fitness levels of target populations. Thus, to improve conservation outcomes of fauna through targeted management strategies, more empirical studies are urgently needed.

### Implications for Biodiversity Conservation

There has been an almost exponential increase in the number of scientific articles published investigating the impacts of winter recreation on fauna since the 1970s. However, at the same time the ski industry has seen a long period of expansion and consolidation [15]. For example, Japan has opened more than 450 new ski fields across the country since 1970, averaging more than 100 new ski fields per decade [50]. Therefore, while the increase in publications is positive for biodiversity conservation in alpine and subalpine areas, it is likely that ecological research has not kept pace with the expansion of the ski industry.

Our systematic review and meta-analysis indicates that significant knowledge gaps remain about the impacts of winter recreation on fauna. Globally, ski resorts are being managed with little empirical evidence (only 27 studies provide specific management actions), thus limiting the implementation of ecologically-sound practices (e.g. minimising damage to native vegetation [11]). There are indications that winter recreation is likely to have a negative impact but the available information is far from conclusive. Less intensively studied taxa and countries show inconsistent effects, and the impacts of ski resorts in unstudied regions and on unstudied taxa remain unknown. Thus there is an urgent need for more regional research if biodiversity is to be effectively conserved, as we cannot confidently assume that the effects reported in one set of regional studies (even on the same taxa) will apply elsewhere.

To best direct the research conducted in alpine and subalpine areas, we need to determine what we want from ecosystems. If the aim is to preserve the original community structure of fauna inhabiting areas impacted by ski tourism, we must rethink what we measure. Community composition and dominant species are often altered by disturbances arising from winter recreation [12], [46], [51] and specialist species are sometimes lost, despite “unchanged” overall species richness [14], [46], [52]. Yet, measures like species richness and abundance are the most commonly reported biotic measures (17/41 studies). Reporting measures of richness and abundance may improve study comparability. But to better understand disturbed communities in relation to reference communities, it would be more useful if measures of community composition and species dominance are also recorded [14].

It is also essential that the research in alpine-subalpine ecosystems consider long-term impacts (effects over greater than 10 years; [53]) of ski resorts on fauna. This is because alpine and subalpine vegetation regenerates slowly due to restricted growing seasons [54], [55], so the effects of winter recreation-related disturbances like ski-run construction may take decades to begin to reverse [33], [42], [56], [57]. However, long-term ecological studies are rare (only five studies investigated the effects of development over more than 10 years), thus our knowledge of the cumulative effects of development and the efficacy of management actions is limited.

### Caveats

We present a review of the global, peer-reviewed literature available to date. However, care needs to be taken when drawing conclusions from the results presented for several reasons. Firstly, “grey” literature was not included in this review due to the difficulty in obtaining this literature in a consistent and balanced way. Without the grey literature, areas identified as knowledge gaps, and regions identified as lacking in research output, may be overemphasized. That said, this highlights the fact that all research needs to be published via widely accessible avenues so that current research and management recommendations are available for use by all researchers, practitioners and government bodies.

A second caveat is that the small number of peer-reviewed papers available, the variability in methodologies, taxa studied and inconsistencies in reporting specific measures, precluded a more comprehensive meta-analysis. Thirdly, binomial tests were used to analyse the available data but due to the limited sample sizes, statistical significance could not always be reliably assessed, hence true effects occurring may not be emphasized.

## Supporting Information

Table S1 Systematic Review and Meta-analysis Proforma.
(DOCX)

Table S2 Examples of positive, non-significant and negative effects for the four composite categories.
(DOCX)

Table S3 Comparison of the contribution of seven regions to the peer-reviewed literature investigating the impacts of ski-related disturbances on wildlife, compared with the proportion of all ski areas located in those seven regions (based on approximate numbers of ski areas provided by [38]).
(DOCX)

Table S4 Provision of management recommendations by region and time period. “Y” indicates that a recommendation was provided; “N” indicates that a recommendation was not provided.
(DOCX)

Checklist S1 **PRISMA Checklist.**
(DOC)

Reference List S1 **Publications included in the Systematic Review and Meta-Analysis.**
(DOCX)
