# Supplementary material for: The Effects of Winter Recreation on Alpine and Subalpine Fauna: A Systematic Review and Meta-Analysis
Source: PLoS One. 2013 May 15;8(5):e64282. doi: 10.1371/journal.pone.0064282 (PMC3655029; doi:10.1371/journal.pone.0064282)
Supplement: Table S1 — Systematic Review and Meta-analysis Proforma. (DOCX) [file pone.0064282.s001.docx]

***Table S1.* Systematic Review and Meta-analysis Proforma.**

| **Datum to be Extracted** | **Description of datum** |
| --- | --- |
| **Author(s)** | *List the authors included on the publication* |
| **Journal** | *Name of journal article was published in* |
| **Year Published** | *Year of article publication* |
| **Year(s) Studied** | *Year(s) actual investigation was carried out* |
| **Time to Publish** | *Time taken for study to be published (numbers of years between last year of data collection to year of publication)* |
| **Location(s)** | *List the specific location(s) where the study was carried out* |
| **Country** | *List the specific country (or countries) where the study was carried out* |
| **Continent** | *Identify the continent where study was carried out* |
| **Species or Community?** | *Was a specific species studied or a faunal community? S/C* |
| **Number of Species Studied** | *How many species were studied?* |
| **Species Studied** | *List all the species studied (where possible)* |
| **Taxonomic Group(s)** | *List the broader taxonomic group(s) studied* |
| **Justification for Study** | *Identify the justification for the study where possible (brief)* |
| **Specific Ski-Related Disturbance** | *List the specific ski-related disturbances studied where possible (e.g. vegetation removal, use of artificial snow etc.)* |
| **General Ski Infrastructure** | *Classify and list the specific ski-related disturbances studied as related to “Ski Runs”, “Resort Infrastructure” and/or “Winter Tourism/Human Disturbance”* |
| **Study Quality** | *Modified from* Felton et al. (2010)* *and* Pullin & Knight (2003)**.  *Studies were assigned to one of four categories of quality:*   1. *A controlled trial with matched pairs of treatments and controls. Scale of replication is suitable for subject taxa.* 2. *Unpaired treatments and controls. Scale of replication is suitable for subject taxa.* 3. *Unpaired treatments and controls. Scale of replication for study raises potential of confounding effects for subject taxa.* 4. *Inherent problems in methodology and/or experimental design.* |
| **Biotic Measure(s) Reported** | *List each of the biotic measures reported in the study* |
| **Composite Category Reported** | *Assign each biotic measure reported in the study to a composite category: Population and Community Measure, Population Viability Measure, Fitness Measure, or ‘Other’ Measure* |
| **Statistic Provided (and/or significance level)?** | *Is a statistic or significance level provided for any of the biotic measures reported? Y/N (Record the data and significance level where possible)* |
| **Overall Effect Concluded** | *Identify the overall effect of the ski-related disturbance on fauna (as concluded by the authors): Positive, Negative, No Effect or Varies (i.e. multiple effects concluded)* |
| **Specific Effects Recorded** | *Identify the specific effect (Positive, Negative or No Effect) recorded for each biotic measure reported in the study* |
| **Specific Management Action Provided?** | *Is a specific management action identified by the authors? Y/N* |

*Felton A, Knight E, Wood J, Zammit C, Lindenmayer D (2010) A meta-analysis of fauna and flora species richness and abundance in plantations and pasture lands. Biol Conserv 143: 545-554.

**Pullin AS, Knight TM (2003) Support for decision making in conservation practice: An evidence-based approach. J Nat Conserv 11: 83-90.
