# Supplementary material for: The Effects of Winter Recreation on Alpine and Subalpine Fauna: A Systematic Review and Meta-Analysis
Source: PLoS One. 2013 May 15;8(5):e64282. doi: 10.1371/journal.pone.0064282 (PMC3655029; doi:10.1371/journal.pone.0064282)
Supplement: Table S2 — Examples of positive, non-significant and negative effects for the four composite categories. (DOCX) [file pone.0064282.s002.docx]

***Table S2.* Examples of positive, non-significant and negative effects for the four composite categories.**

| **Composite Category** | **Negative Effect** | **No Effect** | **Positive Effect** |
| --- | --- | --- | --- |
| **Population & Community Descriptors** |  |  |  |
| Abundance, Biomass and Density | Decrease in abundance, biomass or density in disturbed areas | No significant difference in abundance, biomass or density between disturbed and undisturbed areas | Increase in abundance, biomass or density in disturbed areas |
| Richness and Diversity | Decrease in richness, evenness or diversity in disturbed areas | No significant difference in richness, evenness or diversity between disturbed and undisturbed areas | Increase in richness, evenness or diversity in disturbed areas |
| **Population Viability Measures** |  |  |  |
| Breeding Success | Decrease in breeding success in disturbed areas | No significant difference in breeding success between disturbed and undisturbed areas | Increase in breeding success in disturbed areas |
| Number of Nests | Decreased number of available nests in disturbed areas | No significant difference in number of available nests between disturbed and undisturbed areas | Increased number of available nests in disturbed areas |
| Survival | Decrease in survival rate in disturbed areas | No significant difference in survival rate between disturbed and undisturbed areas | Increase in survival rate in disturbed areas |
| **Fitness Measures** |  |  |  |
| Body Condition | Decreased body condition in disturbed areas | No significant difference in body condition between disturbed and undisturbed areas | Increased body condition in disturbed areas |
| Parasite Load | Increased parasite load in disturbed areas | No significant difference in parasite load between disturbed and undisturbed areas | Decreased parasite load in disturbed areas |
| Sprint Speed | Increased sprint speed in disturbed areas | No significant difference in sprint speed between disturbed and undisturbed areas | Decreased sprint speed in disturbed areas |
| Stress Hormone | Increased stress hormone levels in disturbed areas | No significant difference in stress hormone levels between disturbed and undisturbed areas | Decreased stress hormone levels in disturbed areas |
| **Other Measures** |  |  |  |
| Habitat Use | Decreased use of habitat in areas affected by ski resorts | No significant difference in habitat use inside and outside of areas affected by ski resorts | Increased use of habitat in areas affected by ski resorts |
| Frequency of Occurrence | Decreased frequency of occurrence in disturbed areas | No significant difference in frequency of occurrence between disturbed and undisturbed areas | Increased frequency of occurrence in disturbed areas |

Examples of results we classified as ‘negative’, ‘non-significant’ (no effect) and ‘positive’ for biotic measures reported in the articles we included for review. Biotic measures (hence their reported effects) were subsequently sorted into four composite categories (population and community descriptors, population viability measures, fitness measures and other measures) for analysis.
