# Supplementary material for: The Effects of Winter Recreation on Alpine and Subalpine Fauna: A Systematic Review and Meta-Analysis
Source: PLoS One. 2013 May 15;8(5):e64282. doi: 10.1371/journal.pone.0064282 (PMC3655029; doi:10.1371/journal.pone.0064282)
Supplement: Table S4 — Provision of management recommendations by region and time period. “Y” indicates that a recommendation was provided; “N” indicates that a recommendation was not provided. (DOCX) [file pone.0064282.s004.docx]

***Table S4.* Provision of management recommendations by region and time period.** “Y” indicates that a recommendation was provided; “N” indicates that a recommendation was not provided.

| **Year** | **Australia** | **North America** | **Europe** |
| --- | --- | --- | --- |
| 1970-1979 | - | - | 1Y |
| 1980-1989 | 1Y | - | 1Y, 2N |
| 1990-1999 | - | 2Y | 2N |
| 2000-2009 | 3Y, 1N | 3Y, 2N | 10Y, 6N |
| 2010-present | - | 1Y | 5Y, 1N |
