# Supplementary material for: The Effects of Winter Recreation on Alpine and Subalpine Fauna: A Systematic Review and Meta-Analysis
Source: PLoS One. 2013 May 15;8(5):e64282. doi: 10.1371/journal.pone.0064282 (PMC3655029; doi:10.1371/journal.pone.0064282)
Supplement: Reference List S1 — Publications included in the Systematic Review and Meta-Analysis. (DOCX) [file pone.0064282.s006.docx]

***Reference List S1.* Publications included in the Systematic Review and Meta-Analysis.**

| Amo, L., Lopez, P., Martin, J., 2007. Habitat deterioration affects body condition of lizards: A behavioral approach with *Iberolacerta cyreni* lizards inhabiting ski resorts. Biological Conservation 135, 77-85. |
| --- |
| Arlettaz, R., Patthey, P., Baltic, M., Leu, T., Schaub, M., Palme, R., Jenni-Eiermann, S., 2007. Spreading free-riding snow sports represent a novel serious threat for wildlife. Proceedings of the Royal Society of London, Series B: Biological Sciences 274, 1219-1224. |
| *Ballenger, N., Ortega, C.P., 2001. Effects of ski resort fragmentation on wintering birds in southwest Colorado. Journal of the Colorado Field Ornithologists 35, 122-128. |
| Baratti, M., Migliorini, M., Bernini, F., 2000. Effetti dell'innevamento artificiale sugli Acari oribatei (Acari, Oribatida) delle piste sciabili del Monte Bondone (Trentino, Italia). Studi Trentini di Scienze Naturali Acta Biologica 75, 147-159. |
| Braunisch, V., Patthey, P., Arlettaz, R.L., 2011. Spatially explicit modeling of conflict zones between wildlife and snow sports: Prioritizing areas for winter refuges. Ecological Applications 21, 955-967. |
| Broome, L.S., 2001. Intersite differences in population demography of mountain pygmy-possums *Burramys parvus* Broom (1986-1998): Implications for metapopulation conservation and ski resorts in Koskiuszko National Park, Australia. Biological Conservation 102, 309-323. |
| *Caprio, E., Chamberlain, D.E., Isaia, M., Rolando, A., 2011. Landscape changes caused by high altitude ski-pistes affect bird species richness and distribution in the Alps. Biological Conservation 144, 2958-2967. |
| Caravello, G., Crescini, E., Tarocco, S., Palmeri, F., 2006. Environmental modifications induced by the practice of "artificial snow-making" in the Obereggen/Val d'Ega area (Italy). Journal of Mediterranean Ecology 7, 31-39. |
| Foissner, W., Franz, H., Adam, H., 1982. Terrestrische protozoen als bioindikatoren im boden einer planierten ski-piste. Pedobiologia 24, 45-56. |
| Goldstein, M.I., Poe, A.J., Suring, L.H., Nielson, R.M., McDonald, T.L., 2010. Brown Bear Den Habitat and Winter Recreation in South-Central Alaska. Journal of Wildlife Management 74, 35-42. |
| Goodrich, J.M., Berger, J., 1994. Winter recreation and hibernating black bears *Ursus americanus*. Biological Conservation 67, 105-110. |
| Green, K., 2000. Small mammal activity on the snow surface. Victorian Naturalist 117, 214-218. |
| Hadley, G.L., Wilson, K.R., 2004a. Patterns of density and survival in small mammals in ski runs and adjacent forest patches. Journal of Wildlife Management 68, 288-298. |
| Hadley, G.L., Wilson, K.R., 2004b. Patterns of small mammal density and survival following ski-run development. Journal of Mammalogy 85, 97-104. |
| Haslett, J.R., 1991. Habitat deterioration on ski slopes: Hoverfly assemblages (Diptera: Syrphidae) occurring on skied and unskied subalpine meadows in Austria, In Ravera, O. (Ed.), Terrestrial and Aquatic Ecosystems: Perturbation and Recovery. Ellis Horwood, Chichester, UK, pp. 366-371. |
| Haslett, J.R., 1997. Insect communities and the spatial complexity of mountain habitats. Global Ecology and Biogeography Letters 6, 49-56. |
| Jokimaki, J., Kaisanlahti-Jokimaki, M.L., Huhta, E., Siikamaki, P., 2007. Bird species as indicators of environmental changes at tourist destinations, In Jokimaki, J., Kaisanlahti-Jokimaki, M.L., Tuulentie, S., Laine, K., Uusitalo, M. (Eds.), Environment, Local Society and Sustainable Tourism. University of Lapland, Rovaniemi, Finland, pp. 13-22. |
| *Keßler, T., Cierjacks, A., Ernst, R., Dziock, F., 2012. Direct and indirect effects of ski run management on alpine Orthoptera. Biodiversity and Conservation 21, 281-296. |
| Krebs, J., Lofroth, E.C., Parfitt, I., 2007. Multiscale habitat use by wolverines in British Columbia, Canada. Journal of Wildlife Management 71, 2180-2192. |
| Kübelböck, G., Meyer, E., 1981. Ecological studies of invertebrates in the central high-alps (Obergurgl, Tyrol). VI. Abundance and biomass of Oligochaets (Lumbricidae, Enchytraeidae). Alpin-Biologische Studien 15, 1-52. |
| *Laiolo, P., Rolando, A., 2005. Forest bird diversity and ski-runs: A case of negative edge effect. Animal Conservation 8, 9-16. |
| Lüftenegger, G., Foissner, W., Adam, H., 1986. Der einfluss organischer und mineralischer dunger auf die bodenfauna einer planierten, begrunten schipiste oberhalb der waldgrenze. Zeitschrift fuer Vegetationstechnik 9, 149-153. |
| Mansergh, I.M., Scotts, D.J., 1989. Habitat continuity and social organization of the Mountain Pygmy Possum restored by tunnel. Journal of Wildlife Management 53, 701-707. |
| *Mincheva, Y., Lazarova, S., Peneva, V., 2009. Nematode assemblages from mountain pine (*Pinus mugo* Turra) communities in Pirin Mountain, Bulgaria. Helminthologia 46, 49-58. |
| Morrison, J.R., de Vergie, W.J., Alldredge, A.W., Byrne, A.E., Andree, W.W., 1995. The effects of ski area expansion on elk. Wildlife Society Bulletin 23, 481-481. |
| *Negro, M., Isaia, M., Palestrini, C., Rolando, A., 2009. The impact of forest ski-pistes on diversity of ground-dwelling arthropods and small mammals in the Alps. Biodiversity and Conservation 18, 2799-2821. |
| *Negro, M., Isaia, M., Palestrini, C., Schoenhofer, A., Rolando, A., 2010. The impact of high-altitude ski pistes on ground-dwelling arthropods in the Alps. Biodiversity and Conservation 19, 1853-1870. |
| Nellemann, C., Jordhoy, P., Stoen, O.G., Strand, O., 2000. Cumulative impacts of tourist resorts on wild reindeer (*Rangifer tarandus tarandus*) during winter. Arctic 53, 9-17. |
| Patthey, P., Wirthner, S., Signorell, N., Arlettaz, R., 2008. Impact of outdoor winter sports on the abundance of a key indicator species of alpine ecosystems. Journal of Applied Ecology 45, 1704-1711. |
| Reimers, E., Eftestoel, S., Colman, J.E., 2003. Behavior responses of wild reindeer to direct provocation by a snowmobile or skier. Journal of Wildlife Management 67, 747-754. |
| *Rolando, A., Caprio, E., Rinaldi, E., Ellena, I., 2007. The impact of high-altitude ski-runs on alpine grassland bird communities. Journal of Applied Ecology 44, 210-219. |
| Sanecki, G.M., Green, K., Wood, H., Lindenmayer, D., 2006. The implications of snow-based recreation for small mammals in the subnivean space in south-east Australia. Biological Conservation 129, 511-518. |
| Shine, R., Barrott, E.G., Elphick, M.J., 2002. Some like it hot: Effects of forest clearing on nest temperatures of montane reptiles. Ecology 83, 2808-2815. |
| *Strong, A.M., Dickert, C.A., Bell, R.T., 2002. Ski trail effects on a beetle (Coleoptera: Carabidae, Elateridae) community in Vermont. Journal of Insect Conservation 6, 149-159. |
| Szymkowiak, P., Gorski, G., 2004. Spider communities in the contact zone between open areas and spruce forest in the Karkonosze National Park. Opera Corcontica 41, 309-315. |
| Thiel, D., Menoni, E., Brenot, J.F., Jenni, L., 2007. Effects of recreation and hunting on flushing distance of capercaillie. Journal of Wildlife Management 71, 1784-1792. |
| Thiel, D., Jenni-Eiermann, S., Braunisch, V., Palme, R., Jenni, L., 2008. Ski tourism affects habitat use and evokes a physiological stress response in capercaillie *Tetrao urogallus*: A new methodological approach. Journal of Applied Ecology 45, 845-853. |
| Thiel, D., Jenni-Eiermann, S., Palme, R., Jenni, L., 2011. Winter tourism increases stress hormone levels in the capercaillie *Tetrao urogallus*. Ibis 153, 122-133. |
| Ukkola, M., Helle, P., Huhta, E., Jokimaki, J., Kaisanlahti-Jokimaki, M.L., 2007. The impacts of ski resorts on wildlife in northern Finland, In Jokimaki, J., Kaisanlahti-Jokimaki, M.L., Tuulentie, S., Laine, K., Uusitalo, M. (Eds.), Environment, Local Society and Sustainable Tourism. University of Lapland, Rovaniemi, Finland, pp. 31-41. |
| Watson, A., 1979. Bird and mammal numbers in relation to human impact at ski lifts on Scottish hills. Journal of Applied Ecology 16, 753-764. |
| Watson, A., Moss, R., 2004. Impacts of ski-development on ptarmigan (*Lagopus mutus*) at Cairn Gorm, Scotland. Biological Conservation 116, 267-275. |

***** indicates studies included in the meta-analyses.
