# Supplementary material for: The Effects of Winter Recreation on Alpine and Subalpine Fauna: A Systematic Review and Meta-Analysis
Source: PLoS One. 2013 May 15;8(5):e64282. doi: 10.1371/journal.pone.0064282 (PMC3655029; doi:10.1371/journal.pone.0064282)
Supplement: Table S3 — Comparison of the contribution of seven regions to the peer-reviewed literature investigating the impacts of ski-related disturbances on wildlife, compared with the proportion of all ski areas located in those seven regions (based on approximate numbers of ski areas provided by [38]). (DOCX) [file pone.0064282.s003.docx]

***Table S3.* Comparison of the contribution of seven regions to the peer-reviewed literature investigating the impacts of ski-related disturbances on wildlife, compared with the proportion of all ski areas located in those seven regions (based on approximate numbers of ski areas provided by [38]).**

| **Region** | **Proportion of studies** | **Proportion of all ski areas** |
| --- | --- | --- |
| Australia | 0.12 | 0.002 |
| New Zealand | 0 | 0.005 |
| Asia | 0 | 0.21 |
| South Africa | 0 | 0.0002 |
| South America | 0 | 0.009 |
| North America | 0.20 | 0.16 |
| Europe | 0.68 | 0.62 |
